# Data concerning the proteolytic resistance and oxidative stress in LAN5 cells after treatment with BSA hydrogels

**DOI:** 10.1016/j.dib.2016.08.065

**Published:** 2016-09-06

**Authors:** Pasquale Picone, Giovanna Navarra, Chiara Peres, Marco Contardi, Pier Luigi San Biagio, Marta Di Carlo, Daniela Giacomazza, Valeria Militello

**Affiliations:** a Istituto di Biomedicina e Immunologia Molecolare, Consiglio Nazionale delle Ricerche, Via U. La Malfa 153, 90146 Palermo, Italy; b Dipartimento di Fisica e Chimica, Università di Palermo, Viale delle Scienze, Building 18, 90100 Palermo, Italy; c Istituto di Biofisica, Consiglio Nazionale delle Ricerche, Via U. La Malfa 153, 90146 Palermo, Italy

**Keywords:** Hydrogels, β-aggregates, Cell-scaffold, Drug delivery, Proteolytic resistance, Oxidative Stress

## Abstract

Proteolytic resistance is a relevant aspect to be tested in the formulation of new nanoscale biomaterials. The action of proteolytic enzymes is a very fast process occurring in the range of few minutes. Here, we report data concerning the proteolytic resistance of a heat-set BSA hydrogel obtained after 20-hour incubation at 60 °C prepared at the pH value of 3.9, pH at which the hydrogel presents the highest elastic character with respect to gel formed at pH 5.9 and 7.4 “Heat-and pH-induced BSA conformational changes, hydrogel formation and application as 3D cell scaffold” (G. Navarra, C. Peres, M. Contardi, P. Picone, P.L. San Biagio, M. Di Carlo, D. Giacomazza, V. Militello, 2016) [1]. We show that the BSA hydrogel produced by heating treatment is protected by the action of proteinase K enzyme. Moreover, we show that LAN5 cells cultured in presence of BSA hydrogels formed at pH 3.9, 5.9 and 7.4 did not exhibit any oxidative stress, one of the first and crucial events causing cell death “Are oxidative stress and mitochondrial dysfunction the key players in the neurodegenerative diseases?” (M. Di Carlo, D. Giacomazza, P. Picone, D. Nuzzo, P.L. San Biagio, 2012) [2] “Effect of zinc oxide nanomaterials induced oxidative stress on the p53 pathway” (M.I. Setyawati, C.Y. Tay, D.T. Leaong, 2013) [3].

**Specification Table**

**Table:** 

Subject area	*Physics, Biology,*
More specific subject area	*Protein aggregation*
Type of data	*Figures*
How data was acquired	*Centrifugation by filter and spectrophotometric and DCFH-DA fluorescence measurements*
Data format	*Raw, analyzed*
Experimental factors	*The BSA samples have been incubated 20 h at* 60 °C
Experimental features	*The BSA hydrogel formed after thermal incubation have been tested against proteolytic cleavage and on their ability to induce oxidative stress in LAN5 cell line.*
Data source location	*Palermo (Italy)*
Data accessibility	*Data are provided with this article*

**Value of the data**

• Very important for the protein scaffold is the degradation by proteolytic enzymes.
• LAN5 cell line cultured in the presence of BSA hydrogels at different pH do not show any oxidative stress.
• Present data can help to generate new forms of nanoscale biomaterials based on the protein fibrillar architecture.

## 1 Data

**Fig. 1:**
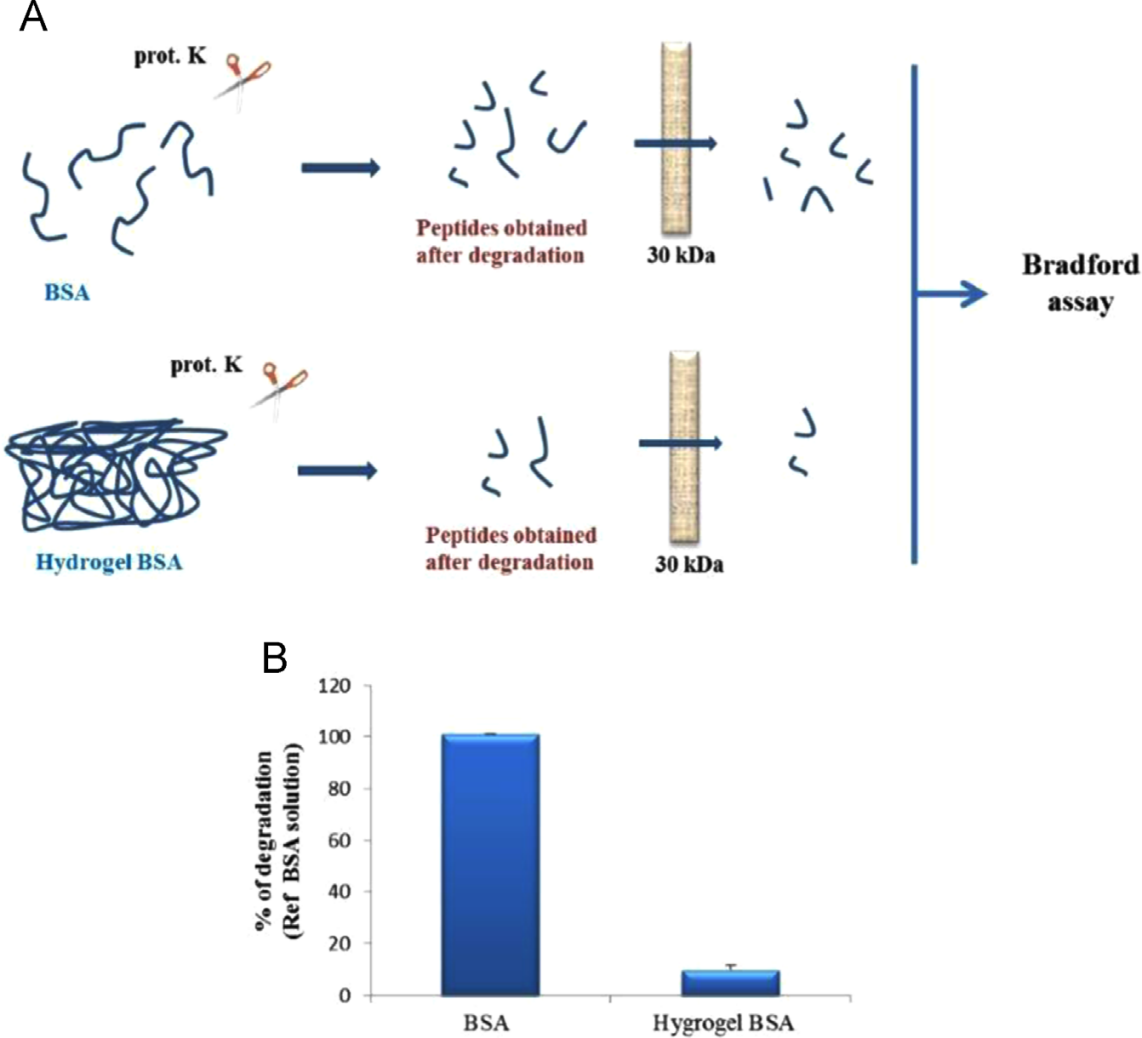
BSA hydrogel resistance to the protease degradation. A) Schematic representation of a model of proteinase K (prot. K) in vitro assay. B) BSA solution and BSA hydrogel were incubated with proteinase K. After filtration, the peptide fragments released were quantified.

**Fig. 2:**
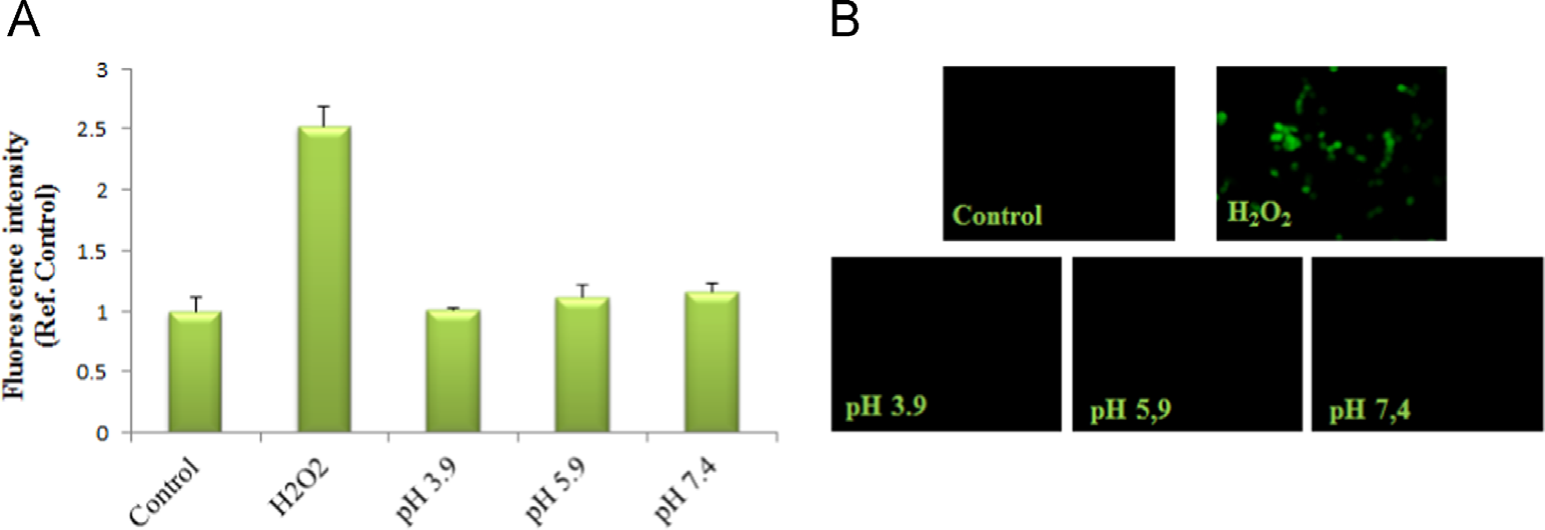
BSA hydrogels do not activate toxic oxidative stress in LAN5 cells. (A) Histogram of DCFH-DA assay represents the green fluorescence intensity with respect to the control. B) Green fluorescent microscopic images.

BSA hydrogel obtained after thermal incubation (60 °C) at pH 3.9 [1] was incubated with proteinase K, an enzyme largely used for protein degradation assay. After filtration, the little peptide fragments released after BSA degradation were quantified by Bradford assay (Fig. 1A). The proteinase K treatment causes about a 10% of degradation of BSA hydrogel with respect to the BSA solution (Fig. 1B), indicating that BSA hydrogel produced by heating treatment is protected against protease degradative attack. Furthermore, the occurrence of oxidative stress [2], [3] due to BSA hydrogels formed at pH 3.9, 5.9 and 7.4 was tested by DCFH-DA assay. Fluorescence data indicated that in BSA hydrogel treated samples the presence of intracellular ROS was comparable to basal levels. In contrast, increased fluorescence was obtained in H_2_O_2_ treated sample used as positive control (Fig. 2A, B). Data clearly showed that no cellular oxidative stress was triggered by our gels.

## 2 Experimental design, materials and methods

The samples, BSA solution and BSA hydrogels obtained after 20-hour incubation at 60 °C, were incubated with proteinase K (25 µg/ml) for 1 h. Then the samples were centrifuged with a centrifugal filter with a pore size of 30 kDa MWCO. The solutions obtained were submitted to Bradford assay and used as suggested by manufacturer (Biorad). Spectroscopic measurements indicate that the BSA hydrogel is protected by proteolytic degradation. Results were expressed as percentage of degradation with respect to the BSA solution.

To assess ROS generation by fluorimeter analysis, the Human neuroblastoma LAN5 cells were plated in a 96-well optical bottom white microplate, while to the microscope fluorescence analysis in a 96-well transparent plate, at the concentrations of 6×10^5^ cell/mL. After the treatment, cells were incubated with 1 µM dichlorofluorescein diacetate (DCFH-DA) in PBS for 10 min at room temperature in the dark. The conversion of non-fluorescent DCFH-DA to the highly fluorescent compound 20,70-dichlorofluorescein (DCF) by cellular esterase activity can be used to monitor the presence of peroxides due to the oxidative burst in the cells. Therefore, the emitted fluorescence is directly proportional to the concentration of hydrogen peroxide inside the cell. After washing in PBS the cells were analyzed by fluorimeter (Microplate reader WallacVictor 2 1420 Multilabel Counter; PerkinElmer, Inc.) and fluorescence microscope (Zeiss Axio Scope 2). The excitation filter was set at 485 nm and the emission filter was set at 530 nm [4].
