# Supplementary material for: Data concerning the proteolytic resistance and oxidative stress in LAN5 cells after treatment with BSA hydrogels
Source: Data Brief. 2016 Sep 6;9:324–7. doi: 10.1016/j.dib.2016.08.065 (PMC5030333; doi:10.1016/j.dib.2016.08.065)
Supplement: Supplementary file 1 — Supplementary material [file mmc1.docx]

**Conflicts of interest**

We confirm that the manuscript “Data concerning the proteolytic resistance and oxidative stress in LAN5 cells after treatment with BSA hydrogels” has been read and approved by all authors and that there are no known conflicts of interest associated with this publication

August 26, 2016.

Picone Pasquale
